# RACE-ETHNICITY, GENDER, AND DISABILITY: A TEST OF STRESS DISABLEMENT PROCESS MODEL AND INTERSECTIONALITY THEORY

**DOI:** 10.1093/geroni/igz038.3019

**Published:** 2019-11-08

**Authors:** Linda A Wray, Linda A Wray, Amy D Thierry

**Affiliations:** 1 Pennsylvania State University, University Park PA, United States; 2 Pennsylvania State University, University Park, Pennsylvania, United States; 3 Xavier University, New Orleans, Louisiana, United States

## Abstract

Decades of studies demonstrate that: physical disability differs widely by race/ethnicity, sex, and achieved social status in midlife and older adults; health conditions explain part or all of the differences by race/ethnicity and/or sex; but disparities by race/ethnicity and/or sex often persist beyond controls for health conditions. Using longitudinal HRS data on 16,280 adults and sequential logistic regression models, Wray and Thierry test if those links are additive or interactive and if achieved social status attenuates remaining disparities. Initial models support past studies indicating that impairments in strength, mobility, and ADLs are greater for females and race/ethnic minority adults. Fully adjusted models, including terms for the intersection of race/ethnicity, sex, and health conditions and controlling for other health and social status factors, reveal differential impacts on reported impairments; and that achieved social status only partly attenuates the disparities. More research is warranted to understand underlying mechanisms that produce health disparities.

